# Computed tomography findings in a Brazilian cohort of 48 patients with pneumonia due to coronavirus disease

**DOI:** 10.1590/0037-8682-0405-2020

**Published:** 2020-07-20

**Authors:** Gabriel Madeira Werberich, Edson Marchiori, Miriam Menna Barreto, Rosana Souza Rodrigues

**Affiliations:** 1 Universidade Federal do Rio de Janeiro, Rio de Janeiro, RJ, Brasil.; 2 Instituto D’Or de Pesquisa e Ensino, Rio de Janeiro, RJ, Brasil.

**Keywords:** COVID-19, Viral infection, Viral pneumonia, Computed tomography

## Abstract

**INTRODUCTION:**

This study was conducted to retrospectively review chest computed tomography (CT) findings in a Brazilian cohort of patients with pneumonia caused by the severe acute respiratory syndrome coronavirus 2 (SARS-CoV-2).

**METHODS:**

Chest CT scans of 78 patients with confirmed coronavirus disease (COVID-19), obtained in March and April 2020, were reviewed. Of 78 cases, the CT scans of 48 (61.5%) showed lung opacities. CT opacity features, their distribution, and the extent of infiltration were evaluated.

**RESULTS:**

The most common CT findings were ground-glass opacities (97.9%), crazy-paving pattern (58.3%), and mixed pattern (18.8%). Rounded lung opacities were observed most frequently (70.8%). Other findings were cystic airspace changes (37.5%), vascular dilatation (35.4%), and the organizing pneumonia pattern (14.6%). The findings were frequently bilateral (87.5%), symmetrical (68.9%), and peripheral (60.2%).

**CONCLUSIONS:**

The most common CT findings were ground-glass opacities and the crazy-paving pattern. Involvement was mostly bilateral, symmetrical, and peripheral. Round opacity morphology was frequently observed and might have some degree of specificity to viral COVID-19 pneumonia.

## INTRODUCTION

Since late December 2019, humanity has been facing a novel coronavirus pandemic caused by the severe acute respiratory syndrome coronavirus 2 (SARS-CoV-2), which originated in Wuhan, China^1^ ^-^ ^3^, and spread worldwide from person to person by means of droplets, direct contact, and inhaled air^4^.The disease caused by SARS-CoV-2 is named coronavirus disease (COVID-19), and it primarily presents as viral pneumonia, with fever, cough, dyspnea, and myalgia^3^. About one-third of patients with COVID-19 develop acute respiratory distress syndrome^5^.

The diagnostic confirmation of SARS-CoV-2 is made by reverse-transcriptase polymerase chain reaction (RT-PCR), but, given the shortage of testing kits and delay in diagnosis, some researchers have suggested that, owing to its high sensitivity (89-97%), chest computed tomography (CT) could be a useful diagnostic tool^6^ ^-^ ^8^. More recent studies have yielded less optimistic results^9^ ^,^ ^10^, and a meta-analysis^11^has shown that the use of chest CT for primary COVID-19 screening or diagnosis would not be beneficial in low-prevalence regions due to substantially low predictive values. Following the publication of these data, several medical societies have suggested that chest CT evaluation be reserved for more severe clinical forms of the disease and for alternative diagnoses^12^ ^-^ ^14^.

Although the role of chest CT in the context of COVID-19 remains under debate and requires further investigation, it is an important imaging tool used for lung parenchyma evaluation, and physicians should be familiar with the most common CT patterns of this disease. Thus, the aims of this study were to identify and describe the common CT features of COVID-19 pneumonia in a large series of Brazilian patients and to compare these with the global findings.

## METHODS

### Patients

Seventy-eight patients who were diagnosed with COVID-19 at our institution (a private hospital in Rio de Janeiro, Brazil) were included in the study. We analyzed only the chest CT scan taken on admission (March or April 2020) for each patient. The institution’s research ethics committees approved the study (CAAE: 29496920.8.0000.5262), and all the patients provided written informed consent.

The inclusion criteria were as follows: 1) age ≥ 18 years; 2) oropharyngeal swab positivity for SARS-CoV-2, determined by RT-PCR; and 3) at least one CT scan showing lung abnormalities. The exclusion criteria were as follows: 1) concomitant, confirmed alternative diagnosis based on chest CT features and 2) incomplete chest CT scan or presence of severe motion artifacts.

### Chest CT protocol

All patients were imaged using a 128-channel multislice dual-source CT system (Somatom Definition Flash, Siemens, Forchheim, Germany). Imaging was done at end inspiration with the patient in the supine position, from the lung apices down to the costophrenic angles, at 120 kV and modulated milliamperes (120 to 300 mA). 

After the acquisition, all images were reconstructed with 1.5 mm slice thickness using a high spatial frequency reconstruction algorithm and stored in the PACS system. All images were viewed on both parenchymal (width: 1500 HU, level: -700 HU) and mediastinal (width: 350 HU, level: 40 HU) settings.

### Image analysis

Two chest radiologists with 23 and 25 years of experience, respectively, independently evaluated the CT images using the criteria defined in the Fleischner Society’s Glossary of Terms^15^. They identified and recorded the following: 1) ground-glass opacities (hazy areas of increased attenuation without obscuration of the underlying vasculature), 2) consolidation (homogeneous opacification with obscuration of the underlying vasculature; parenchymal consolidation was characterized when the area exceeded 3 cm), 3) mixed pattern (consolidation area < 3 cm with ground-glass opacity), 4) crazy-paving pattern (intra- or interlobular lines superimposed over ground-glass opacity), 5) opacities with rounded morphology (ground-glass opacity, consolidation, mixed or crazy-paving pattern), 6) reversed halo sign (a focal rounded area of ground-glass opacity, with or without reticulation, surrounded by a more or less complete ring of consolidation), 7) halo sign (ground-glass opacity surrounding a nodule), 8) interlobular septal thickening, 9) reticular pattern and subpleural lines (coarse, irregular linear/curvilinear opacities associated with architectural distortion or perilobular thickening), 10) patterns resembling organizing pneumonia and chronicity, and 11) airway abnormalities (bronchial wall thickening, bronchiectasis, and endoluminal secretion). The evaluators also assessed the presence of cystic air spaces in areas with infiltrate, in subpleural regions, and along the peribronchovascular interstitium. Finally, the dominant pattern was recorded. The presence of pleural effusion, pericardial effusion, thoracic lymphadenopathy (defined as lymph node short axis ≥ 10 mm), and underlying lung disease (such as emphysema or fibrosis) were also noted.

The predominant distribution of disease (axial, anteroposterior, or craniocaudal) was determined. Axially distributed abnormalities were considered to be predominantly peripheral when they were localized in the peripheral one-third of the lung, predominantly central when they involved mainly the central two-thirds of the lung, or to have no predominance. Symmetry and laterality (uni- or bilateral) were also noted. The number of affected lobes was recorded as one, two, three, or more.

To quantify the extent of parenchymal disease, a semi-quantitative regional scoring system was used^16^. Each lung was divided into upper (above the carina), middle, and lower (below the inferior pulmonary vein) zones. The degree of involvement of each lung zone was scored visually on a scale ranging from 0 to 4 (0: no involvement, 1: <25%, 2: 25-50%, 3: 50-75%, and 4: >75% involvement). Overall CT scores (range: 0-24) were obtained by summing the scores for the six lung zones. Data are presented as frequency and percentage, mean ± standard deviation, and median (interquartile range).

## RESULTS

We analyzed chest CT scans from 78 consecutive patients with swab-proven diagnoses of COVID-19, of whom 48 (61.5%) had abnormal findings. Most (*n* = 34, 71%) patients with abnormal findings were male, and the median age of all patients with abnormal scans was 59.8 years (range: 28-93 years). The patients’ demographic and clinical characteristics and laboratory findings on admission are summarized in Table 1. The periods between symptom onset and CT examination ranged from 1 to 15 days (median: 5.3 days). 

**TABLE 1:** Demographic and clinical characteristics and laboratory findings on admission of the patients.

		Patients (n = 48)
**Sex**	Male	34 (71%)
	Female	14 (29%)
**Median age (range)**		59.8 years (28-93 years)
**Clinical symptoms**	Fever	40 (83%)
	Cough	32 (67%)
	Dyspnea	21 (44%)
	Fatigue	18 (37%)
	Headache	8 (17%)
	Rhinorrhea	7 (14%)
	Diarrhea	5 (10%)
	Anosmia	4 (8%)
	Arthralgia	4 (8%)
**Laboratory findings**	Elevated CRP	40 (83%)
	Lymphopenia	28 (58%)
	Elevated D-dimer	22 (46%)
	Elevated DHL	16 (33%)
	Leukopenia	10 (21%)
	Neutrophilia	9 (19%)
	Leukocytosis	3 (6%)
	Lymphocytosis	1 (2%)

**CRP:** C-reactive protein; **DHL:** dehydrogenase lactate.

**FIGURE 1:**
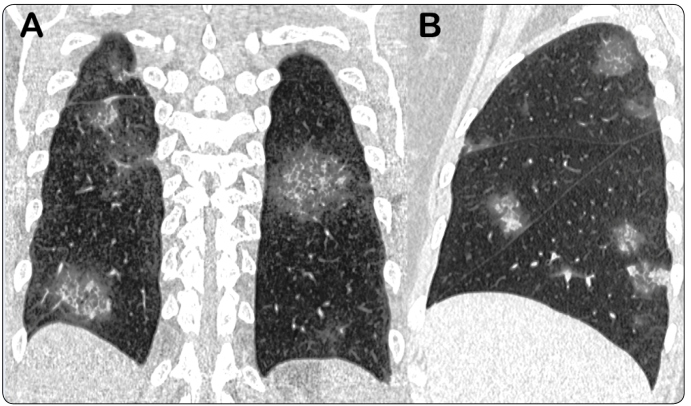
**(A)** Coronal reformatted CT image of a 59-year-old man with COVID-19 presenting with fever and showing multiple bilateral rounded opacities with crazy-paving aspect. **(B)** Sagittal reconstructed chest CT image of a 52-year-old woman with COVID-19 pneumonia showing multifocal, patchy, rounded ground-glass and mixed opacities.

**FIGURE 2:**
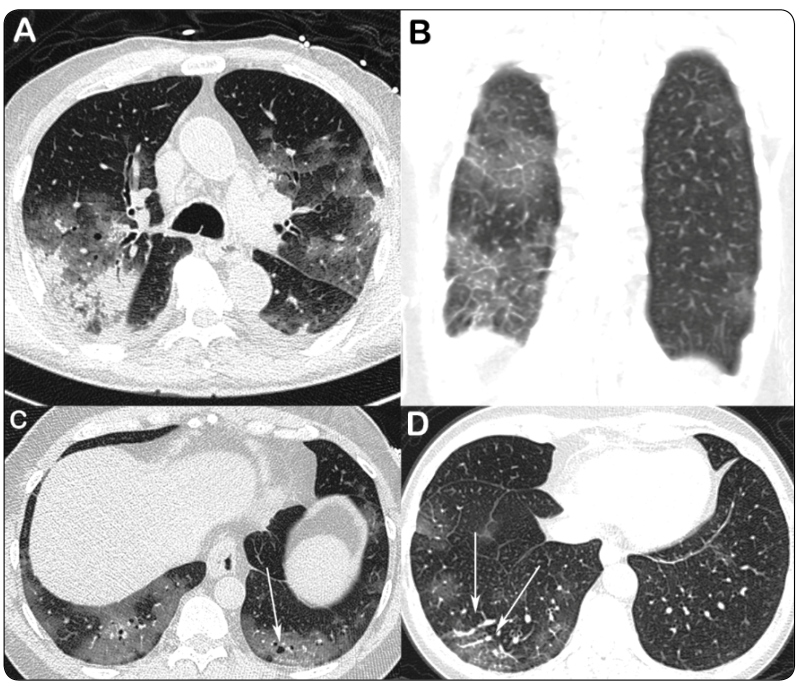
CT images obtained from four different patients with COVID-19 pneumonia. **(A)** Axial CT image of an 86-year-old man showing multiple bilateral patchy areas of ground-glass opacity. Also note peripheral areas of parenchymal consolidation in the right upper lobe. **(B)** Coronal reconstructed chest CT image of a 59-year-old man showing interlobular lines superimposed on a background of ground-glass opacity (crazy-paving pattern) in the right lung. **(C)** Axial CT image of a 56 year-old-man depicting tiny cystic air spaces (arrow). **(D)** Axial CT image of a 64-year-old woman showing vascular dilatation associated with ground-glass opacities in the right lower lobe (arrows).

The most frequent abnormal CT finding was ground-glass opacity (*n* = 47, 97.9%), followed by crazy-paving pattern (*n* = 28, 58.3%), mixed pattern (*n* = 9, 18.8%), and consolidation (*n* = 5, 10.4%; Table 2 and Table 3). Rounded lung opacities were observed in 34 (70.8%) patients; these opacities presented as ground-glass opacities, crazy-paving pattern, mixed patterns, and consolidation in 22 (45.8%), 6 (12.5%), 4 (8.3%), and 2 (4.2%) patients, respectively. Vascular dilatation and cystic air spaces were observed in 17 (35.4%) and 18 (37.5%) cases, respectively (Figures 1 and 2). Less common findings were bronchial wall thickening (*n* = 8, 16.7%), the reversed halo sign (*n* = 2, 4.2%), mucus plugs (*n* = 2, 4.2%), and centrilobular nodules (*n* = 2, 4.2%). Septal thickening, reticular pattern, and subpleural lines were observed in 13 (21.3%), 8 (16.7%), and 7 (14.6%) cases, respectively. Pleural effusion, pericardial effusion, and enlarged lymph nodes were seen in two (4.2%), three (6.2%), and two (4.2%) cases, respectively. No halo sign was observed. The dominant CT pattern was ground-glass opacity (*n* =25, 52.1%), followed by crazy-paving pattern (*n* = 12, 25%). A CT pattern of organizing pneumonia (OP) was observed in seven (14.6%) patients. No patient in our study had pulmonary fibrosis.

**TABLE 2:** Frequency of CT findings.

Findings	Frequency n (%)
Ground-glass opacity	47 (97.9%)
Rounded opacity	34 (70.8%)
Crazy-paving pattern	28 (58.3%)
Cystic airspace changes	18 (37.5%)
Vascular dilatation	17 (35.4%)
Mixed pattern	9 (18.8%)
Bronchial wall thickening	8 (16.7%)
Reticular pattern	8 (16.7%)
Organizing pneumonia pattern	7 (14.6%)
Subpleural line	7 (14.6%)
Consolidation	5 (10.4%)
Pericardial effusion	3 (6.2%)
Pleural effusion	2 (4.2%)
Centrilobular nodules	2 (4.2%)
Mucus plug	2 (4.2%)
Reversed halo sign	2 (4.2%)
Enlarged lymph nodes	2 (4.2%)

**TABLE 3:** Spatial distribution of CT findings.

Distribution	Frequency n (%)
**Craniocaudal predominance**	
Lower lobes	22 (45.9%)
Middle lobe	6 (12.5%)
Upper lobes	5 (10.4%)
No predominance	15 (31.2%)
**Anteroposterior dominance**	
Posterior lung aspect	25 (52%)
No predominance	23 (48%)
**Axial predominance**	
Peripheral	30 (60.2%)
Central	1 (2.1%)
No predominance	17 (35.4%)
**Laterality**	
Bilateral	42 (87.5%)
Unilateral	6 (12.5%)
**Distribution**	Frequency n (%)
**Symmetry**	
Symmetric	33 (68.9%)
Right predominance	13 (27%)
Left predominance	2 (4.1%)
**Number of affected lobes**	
1	3 (6.3%)
2	4 (8.3%)
3 or more	41 (85.4%)
**Extent of pulmonary involvement**	
Less than 25%	24 (50%)
25-50%	17 (35.3%)
50-75%	6 (12.6%)
More than 75%	1 (2.1%)

Abnormalities were bilateral, symmetrical, multilobar, predominantly peripheral, in the lower lobes, and in the posterior aspect of the lungs in 42 (87.5%), 33 (68.9%), 41 (85.4%), 30 (62.6%), 22 (45.9%), and 25 (52%) cases, respectively. Subpleural sparing was observed in only three (6.3%) cases. The degree of lung involvement was <25%, 25-50%, 50-75%, and >75% in 24 (50%), 17 (35.3%), 6 (12.6%), and 1 (2.1%) case, respectively.

In our sample of 48 patients with abnormal CT findings, five (10.4%) patients died (age: 78 to 88 years; median: 85 years). Of these, two (40%) patients exhibited more than 50% lung involvement. Among the 43 patients who did not die, 11 (25.6%) patients exhibited more than 50% lung involvement. Twenty nine (60.4%) patients received only clinical treatment or non-invasive oxygen support, 19 (39.6%) were admitted to the intensive care unit, and 14 (73.7%) of them needed mechanical ventilation for supportive care. Thirty patients with normal CT findings were discharged from the hospital.

## DISCUSSION

In this study sample of 78 symptomatic patients with confirmed SARS-CoV-2 infection, 48 (61.5%) patients exhibited lung opacities on CT. The most common clinical presentation at admission entailed fever, cough, and dyspnea, consistent with previous reports^3^ ^,^ ^5^ ^,^ ^6^ ^,^ ^17^. All selected patients had abnormal chest CT findings, indicative of COVID-19 pneumonia. The most frequently observed lung CT features in patients with COVID-19 pneumonia included ground-glass opacities, followed by crazy-paving and mixed patterns, which was in agreement with previous reports^5^ ^,^ ^18^ ^-^ ^21^. Although our results were in accordance with those from previous studies, we highlight several peculiarities. In a systematic review of 919 patients, Salehi et al.^22^reported that the frequency of consolidation was 31%, which was higher than that observed in our sample. They also reported a high frequency of ground-glass opacities, similar to that observed in our sample. The low frequency of consolidation observed in this study may be attributed to the separate consideration given to larger areas (>3.0 cm) of parenchymal consolidation and mixed opacity. Relative to our findings, Chung et al.^19^found lower frequencies of ground-glass opacity (57%), crazy-paving pattern (19%), and peripheral distribution (33%) in a sample of 21 patients. Similarly, Zhu et al.^23^ found a lower frequency of ground-glass opacities (47%). Our results were similar to those of Song et al.^24^, who described a predominance of ground-glass opacities (77%) in their study sample. Few reports have described the morphology of these lung opacities. We observed rounded lung opacities in 70.8% patients, whereas Zhou et al.^25^ and Chung et al.^19^ found them in only 25.8% and 33% of patients, respectively. A consensus statement suggested that rounded lung opacities should be considered as a typical COVID-19 feature on CT scans^26^.

Another characteristic aspect described in the literature is vascular enlargement, found in 35.4% of our patients. Bai et al.^27^ reported vascular enlargement in 59% of patients with COVID-19 pneumonia and in 22% of those with non-viral pneumonia. This aspect is believed to be caused by proinflammatory factors^28^. We observed mediastinal lymph node enlargement in only 4.2% of our patients, in accordance with the observations from many Chinese series^19^ ^-^ ^21^ ^,^ ^25^ ^,^ ^28^ ^,^ ^31^ ^,^ ^32^ but contrasting the higher frequencies observed in two European cohorts (66% in a French study^29^and 58% in an Italian study^30^). Airway disease, solid pulmonary nodules, cavitation, and pleural effusion were infrequent observations in our sample, a finding in accordance with the results of previous studies^16^ ^,^ ^19^ ^-^ ^21^ ^,^ ^25^ ^,^ ^28^ ^,^ ^31^ ^,^ ^32^.

We observed cystic changes in about one-third of our patients. Previously, few reports have described these features^8^ ^,^ ^28^ ^,^ ^31^. The pathophysiology of cystic air space development in COVID-19 patients is unclear. Some authors^29^ ^,^ ^32^ have suggested that they are associated with the repair process, and Zhou et al.^25^ hypothesized that these spaces represent the development of small pneumatoceles during the healing phase.

Our observation of the presence of predominantly bilateral, peripheral, multilobar, and lower-lung distributed parenchymal lesions is similar to that reported in previous literature^16^ ^,^ ^19^ ^-^ ^21^ ^,^ ^25^ ^,^ ^28^ ^,^ ^31^ ^,^ ^32^. We observed posterior lung involvement in approximately half of our patients and symmetrical lung abnormalities in 68.9% of patients. No previous study has described such peculiar distribution of lesions in patents with COVID-19 pneumonia.

Our study had some limitations. First, our sample size was small, and although we observed a wide range of features that encompasses those described by other authors, we were not able to correlate the CT findings with patient outcome. Second, we analyzed only chest CT scans acquired at admission, not the findings since the onset of symptoms. Finally, most of the CT findings reported here are nonspecific and may be seen in patients with other infectious and non-infectious diseases. Although we excluded patients with confirmed alternative diagnoses, the CT findings in the recruited cases could have be attributed to other causes, such as concomitant infection. However, since biopsy of such cases is not possible, this limitation is commonly seen in other similar studies.

In summary, knowledge of the most frequent CT findings in COVID-19 pneumonia patients can provide valuable information for physicians. Our data are in agreement with those reported in the literature to date and showed that the peripheral distribution of bilateral, multifocal ground-glass opacities is the imaging hallmark of this infection. We found rounded lung opacities, cystic changes, and symmetrically distributed lung abnormalities in a large proportion of this Brazilian cohort. This information might aid in the diagnosis of COVID-19 pneumonia.
